# Inhibitory Effects of Verrucarin A on Tunicamycin-Induced ER Stress in FaO Rat Liver Cells

**DOI:** 10.3390/molecules20058988

**Published:** 2015-05-19

**Authors:** Eun Young Bae, Seung Woong Lee, Sin Seong, Wonjun Cho, Jong Seog Ahn, Hyun-Sug Cho

**Affiliations:** 1 LINC Project Group, Daejeon University, Daejeon 300-716, Korea; E-Mail: eunjs75@dju.kr; 2 Department of Chemistry, Mokwon University, Daejeon 302-729, Korea; E-Mail: lswdoc@mokwon.ac.kr; 3 Soram Korean Medicine Hospital, M-Tower, Gangnam-Gu, Seoul 135-879, Korea; E-Mail: ss9335@soram.kr; 4 Soram Bio-Medicine Research Institute (SBRI), Soram Korean Medicine Hospital, M-Tower, Gangnam-Gu, Seoul 135-879, Korea; E-Mail: williamcho86@soram.kr; 5 Chemical Biology Research Center, Korea Research Institute of Bioscience and Biotechnology (KRIBB), Yeongudanjiro, Ochang, Cheongwon, Chungbuk 363-883, Korea; 6 Hyehwa-Liberal Arts College, Daejeon University, Daejeon 300-716, Korea

**Keywords:** verrucarin A, *Fusarium* sp. F060190, tunicamycin, ER stress, FaO rat liver cells

## Abstract

Endoplasmic reticulum (ER) stress is linked with development and maintenance of cancer, and serves as a therapeutic target for treatment of cancer. Verrucarin A, isolated from the broth of *Fusarium* sp. F060190, showed potential inhibitory activity on tunicamycin-induced ER stress in FaO rat liver cells. In addition, the compound decreased tunicamycin-induced GRP78 promoter activity in a dose dependent manner without inducing significant inhibition of luciferase activity and cell growth for 6 and 12 h. Moreover, the compound decreased the expression of *GRP78*, *CHOP*, *XBP-1*, and suppressed XBP-1, and reduced phosphorylation of IRE1α in FaO rat liver cells. This evidence suggests for the first time that verrucarin A inhibited tunicamycin-induced ER stress in FaO rat liver cells.

## 1. Introduction

The endoplasmic reticulum (ER) is an organelle present in all eukaryotic cells, and it plays a leading role in lipid and protein synthesis [1]. Membrane-spanning proteins or hydrosoluble proteins are folded by a chaperone in ER, in a manner parallel with protein synthesis at ER-bounded ribosomes. However, this folding process is disturbed by heat shock, inhibition of sugar chain modification, dysregulation of calcium dynamics, metabolic disorders, viral infections, etc. Under these conditions, proteins are misfolded or unfolded with subsequent aggregation of unusual proteins and apoptosis [2,3]. ER stress is implicated in various diseases such as inflammatory disorders, metabolic disease, and cancer [4,5]. ER stress is caused by various pathological or physiological condition such as ER calcium depletion, hypoxia, oxidative stress, and DNA damage, which can activate unfolded protein response (UPR), followed by restoration from stress conditions. The ER stress response is signaled in part through the dimerization of ER membrane-localized inositol requiring enzyme-1α (IRE1α). IRE1α is a serine-threonine protein kinase and endoribonuclease that, upon activation, initiates the unconventional splicing of the mRNA encoding X-box-binding protein-1 (XBP-1) [6,7,8]. This splicing reaction creates a translational frameshift to produce an active XBP-1 transcription factor. Activated XBP-1 has been reported to induce up-regulation of molecular chaperons such as GRP78 and ERdj4 to diminish the accumulation of unfolded proteins [9]. The 78-kDa glucose-regulated protein GRP78 is a central regulator of endoplasmic reticulum (ER) homeostasis, functioning in protein folding, ER calcium binding and modulation of transmembrane ER stress sensor activity [10]. Mice deficient in XBP-1, a transcription factor that modulates ER stress response, have been reported to develop insulin resistance [11]. Therefore, it has been suggested that candidates with reduced the ER chaperone expression levels, could not only be employed for the treatment of type 2 diabetes and obesity, but also for the treatment of diseases related with dysfunction of ER. Previously, there have been a number of reports on the design and development of synthetic ER stress inhibitors; however there exist only a few reports on ER stress inhibitors derived from fungi.

In the course of our screening program for identifying inhibitors of ER stress-induced XBP-1 activation and regulators of GRP78/Bip molecular chaperone expression using the microbial cultures libraries, we detected potent inhibitory effect in an extract of the soil fungus, *Fusarium* sp. F060190. Assay-guided fractionation of the EtOAc extract led to the isolation of the known compound, verrucarin A (1), from mass cultures of fungus strain *Fusarium* sp. F060190, as ER stress modulator. In the current study, we firstly reported that verrucarin A inhibited the ER stress-induced XBP-1 activation and GRP78/Bip molecular chaperone expression in FaO cells.

## 2. Results and Discussion

### 2.1. Isolation and Identification of Verrucarin A

**Figure 1:**
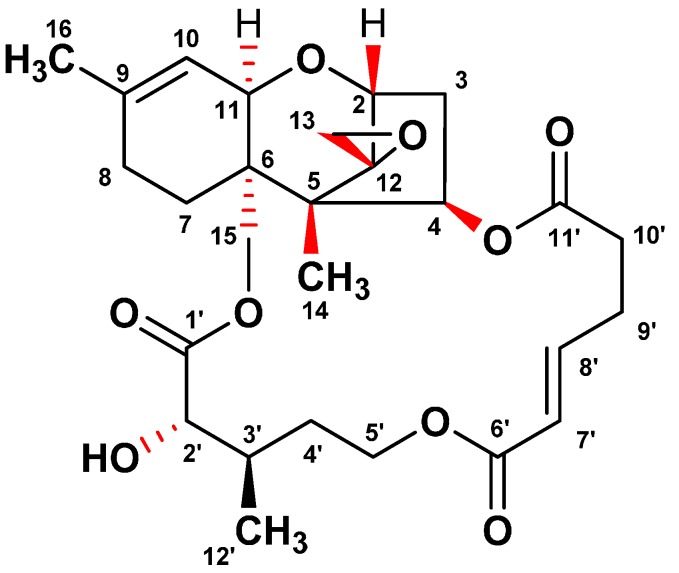
Chemical structure of verrucarin A isolated from the culture broth of *Fusarium* sp. F060190.

In the course of the screening program for identifying ER stress inhibitors from microbial resources, a fungal isolate F060190 was selected based on its potent inhibitory effect against ER stress. To isolate the active compounds with an inhibitory effect on the GRP78 luciferase activity, the culture broth of *Fusarium* sp. F060190 was extracted with EtOAc. The EtOAc extract was separated on silica gel and ODS open-column chromatography, and then subjected to semi-preparative HPLC to yield compound **1**. As shown in Figure 1, the structure of compound **1** was confirmed as verrucarin A (**1**) through NMR and MS spectra analyses, and comparison with published literature data [12,13].

### 2.2. Verrucarin A Inhibits Tunicamycin-Induced GRP78 Expression

GRP78 acts as a molecular chaperone in the endoplasmic reticulum (ER) to promote protein folding [14]. Thus, substances which either down- or up-regulate GRP78 transcription, are expected to be promising drugs for the treatment of type 2 diabetes and obesity [11]. In the course of our screening program for identifying regulators of GRP78 molecular chaperone expression, we isolated a known compound, designated as verrucarin A (Figure 1), which inhibited GRP78 expression in FaO cell reporter gene assay system.

**Figure 2:**
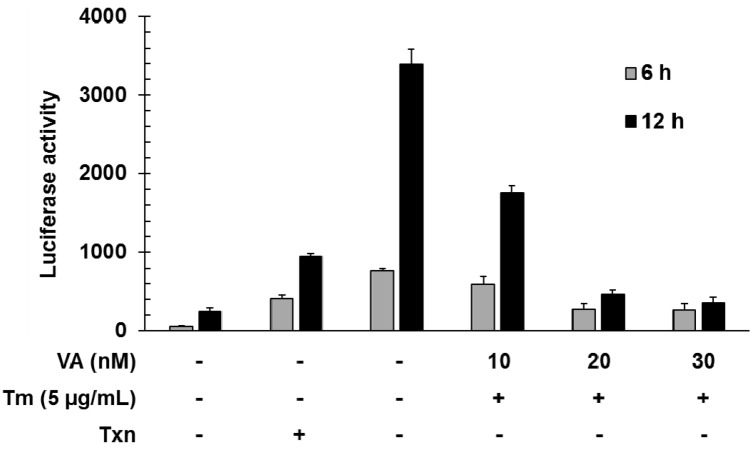
Inhibitory effects of GRP78 induced by verrucarin A in FaO rat liver cells. Cells in 24-well plates were transfected with 0.8 mg of GRP78-Luc for 5 h and then incubated in serum medium. After 24 h, each dose 10, 20, and 30 nM verrucarin A pre-treated for 1 h, and then 5 μg∙mL^−1^ tunicamycin (Tm) treated for 6 and 12 h. Subsequently, the cells were washed with cold PBS and lysed for measurement of luciferase activity. (+): treated with Tm or Txn, (−): untreated with Tm or Txn.

To investigate the function of verrucarin A, we first examined the dose effect of verrucarin A on GRP78 promoter regulation by using a luciferase reporter gene under the control of the GRP78 promoter. Tunicamycin is a well-known as an inhibitor of protein glycosylation and a ER stress inducer. As shown in Figure 2, verrucarin A decreased the expression of the reporter gene at concentrations of 10, 20, and 30 nM when cells were co-stimulated with tunicamycin for 6 and 12 h.

### 2.3. Effect of Verrucarin A on Cell Viability

**Figure 3:**
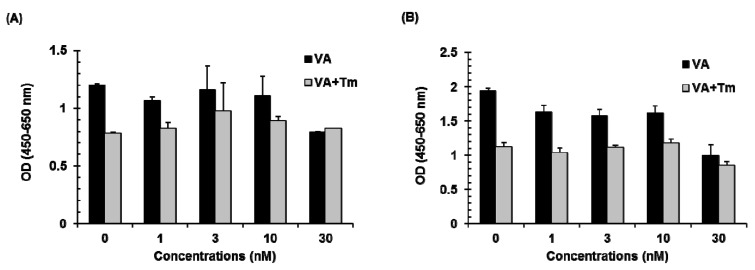
Effect of verrucarin A (VA) on the cell viability of FaO rat liver cells. Cells pre-treated with 1, 3, 10, and 30 nM of verrucarin A or not with each dose for 1h were exposed to 5 μg∙mL^−1^ tunicamycin (Tm) for 6 h (**A**) and 12 h (**B**). Subsequently, cell viability was measured with cell viability assay kit. Bars represent means ± SE from representative triplicate experiments.

The effect of verrucarin A alone or in combination with tunicamycin on cell growth was evaluated in FaO rat liver cells. As shown in Figure 3, verrucarin A showed no significant variations in cell viability as compared with control at 1–10 nM and 5 μg∙mL^−1^ of tunicamycin treatment. However, a significant increase in the cytotoxic effect of verrucarin A was noted at 30 nM as compared to cells treated with tunicamycin at 6 h and 12 h. The result of cell viability assay is presented in Figure 3.

### 2.4. Effect of Verrucarin A on ER Stress-Related Gene Expression

**Figure 4:**
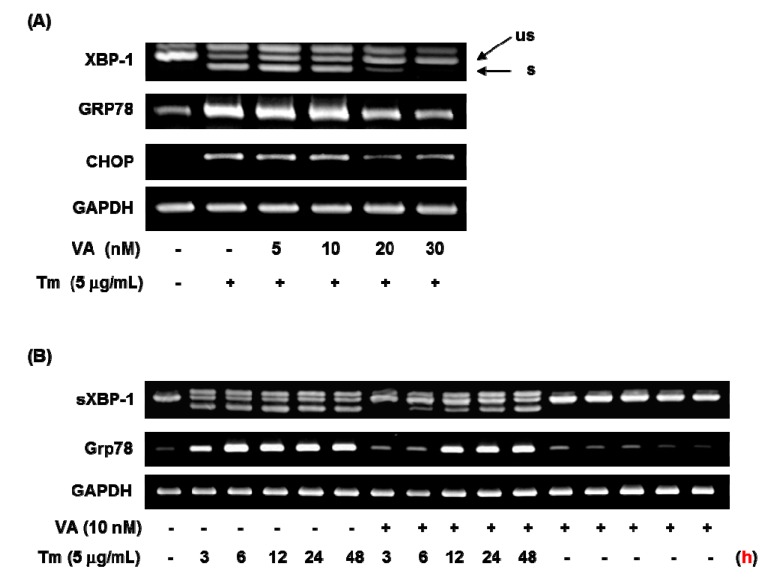
Verrucarin A inhibits ER stress in FaO cells. (**A**,**B**) RT-PCR analysis. Tunicamycin (Tm)-induced XBP-1 splicing, GRP78 and CHOP gene expression were inhibited by verrucarin A. Cells pre-treated with verrucarin A at concentrations of 0, 5, 10, 20, and 30 nM or not with each dose for 1 h were exposed to 5 μg∙mL^−1^ Tm for 6 h in FaO cells. The arrows represent the unspliced form (us) and spliced form (s) of XBP-1. (+): treated with Tm, (−): untreated with.

To investigate the effect of verrucarin A on ER stress-related gene expressions such as GRP78, XBP-1, and CHOP, this was studied by using RT-PCR analysis. As displayed in Figure 4, 5 nM of verrucarin A exhibited effects on GRP78, XBP-1, and CHOP mRNA expression. After 6 h treatment, 20 nM of verrucarin A dramatically reduced mRNA expression of GRP78, XBP-1, and CHOP genes. Accordingly, 30 nM of verrucarin A suppressed tunicamycin-induced XBP-1 splicing, GRP78, and CHOP gene expression after 6 h. These findings suggested that verrucarin A could inhibit ER stress in FaO cell (Figure 4A). However, the expression of GRP78 and XBP-1 after treatment with 10 nM of verrucrin A exhibited a different tendency of downregulation at 3 h and subsequent increase at 12 h as compared with the control (Figure 4B). These data suggest that 10 nM of verrucarin A has a short-term effect on suppression of tunicamycin-induced ER stress. Furthermore, inhibition of the XBP-1 splicing form presence of 10 nM of verrucarin A at 3, 6, and 12 h was noted. Interestingly, 10 nM of verrucarin A suppressed the 3 h treatment of the XBP-1 splicing form (Figure 4B).

**Figure 5:**
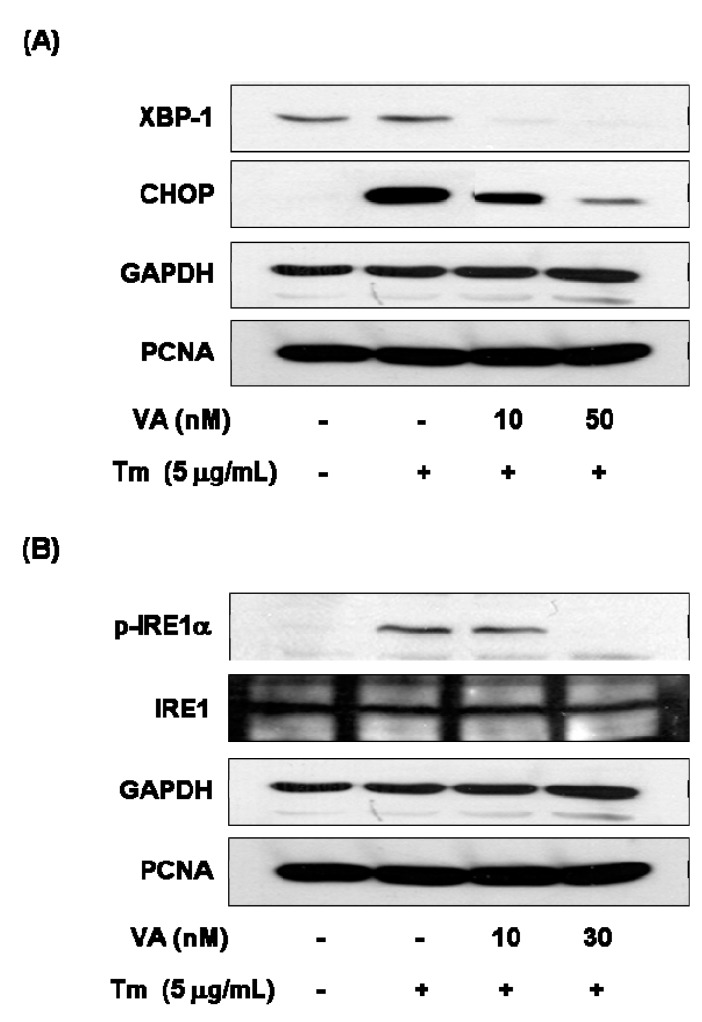
Verrucarin A inhibits ER stress in FaO cells. (**A**,**B**) Western blotting analysis. Tm-induced XBP-1, CHOP, IRE1 expression were inhibited by VA. Cells pre-treated with 0, 10, and 30 nM of VA or not with each dose for 1 h was exposed to 5 μg∙mL^−1^ Tm for 6 h in FaO cells. (+): treated with Tm, (−): untreated with.

In order to determine ER stress-related protein changes, following combination with verrucarin A exposure, western blotting was employed to analyze the expression of CHOP, XBP-1, and p-IRE1α. The protein expression of GRP78 and CHOP exhibited reduction in the presence of 10 nM of verrucarin A (Figure 5A). These results suggest a mechanism by which early response of verrucarin A may inhibit the action of XBP-1 and CHOP expression after 6 h. The analysis also revealed that 30 nM of verrucarin A markedly decreased tunicamycin-induced IRE1α phosphorylation (Figure 5B).

## 3. Experimental Section

### 3.1. Fermentation, Extraction and Isolation of Verrucarin A

*Fusarium* sp. F060190 was cultured in 1 L Erlenmeyer flask, each containing 200 mL of medium containing glucose 20 g, yeast extract 2 g, peptone 5 g, MgSO_4_∙7H_2_O 0.5 g, and KH_2_PO_4_ 1 g in 1 L of distilled water, pH 5.6–5.8. Flasks were individually inoculated with 2 mL seed cultures of *Fusarium* sp. F060190. Flask cultures were incubated at 28 °C and aerated by agitation on a rotary shaker at 140 rpm for a period of 7 days. Extraction of the filtered fermentation broth with EtOAc (5 L) provided an organic phase, which was then concentrated using a rotary evaporator to yield 1.2 g of crude extract. The EtOAc extract was subjected to C18 flash column chromatography (5 × 40 cm), eluting with a stepwise gradient of 20%, 40%, 60%, 80%, and 100% (v/v) MeOH in H_2_O (500 mL each). Among the seventeen pools (Fr.1–Fr.17) combined by their TLC profile, Fr. 8 eluted with 70% MeOH was found to have the highest activity. Thus, Fr. 8 was purified by semi-preparative reversed-phase HPLC eluting with a gradient from 50% to 70% CH_3_CN in H_2_O over 30 min to yield compound **1** (35 mg; t_R_ = 26 min). 

### 3.2. Chemicals

Dulbecco’s modified Eagle’s medium and fetal bovine serum were purchased from Gibco-BRL (Grand Island, NY, USA). Antibodies against CHOP were obtained from Cell Signaling Technology (Beverly, CA, USA) and antibody against p-IRE1α was from Abcam (Cambridge, UK). In addition, antibodies against IRE1 α, XBP-1, GAPDH, and PCNA were purchased from Santa Cruz Biotechnology (Dallas, CA, USA). Enhanced Cell Viability Assay Kit (EZ-CyTox) was obtained from Daeil Lab Service (Seoul, Korea), and luciferase assay kit was from Promega (Madison, WI, USA). Chemiluminescence reagent was obtained from Thermo Scientific (Burlington, IL, USA).

### 3.3. Cell Culture and Transfection

FaO rat liver cells were maintained in Dulbecco’s modified Eagle’s medium supplemented with 2 mM L-glutamine, and 10% heat-inactivated fetal bovine serum, and were cultured in a humidified CO_2_ incubator at 37 °C. For luciferase reporter assays, cells (80%–90% confluence) were incubated in 12-well plates for 24 h. Using Lipofectamine 2000 (Invitrogen, Carlsbad, CA, USA), cells were subsequently transfected with 0.8 µg of pGRP78-luc. After 24 h, cells were treated with verrucarin A and tunicamycin (Sigma-Aldrich, St. Louis, MO, USA) for various times and luciferase activity was measured with a detection kit.

### 3.4. Cell Viability Assay

For the cell viability assay, semi-confluent cells in a 96-well plate were treated with tunicamycin and/or verrucarin A. After adding 10 µL of the EZ-CyTox solution to each well of the culture plate for 2 h. The absorbance was measured using a micro-plate reader at 450 nm (reference wavelength at 650 nm).

### 3.5. Semiquantitative RT-PCR Experiments

For RT-PCR, total RNA was harvested from cells using Tri-Reagent (MRC, Cincinnati, CA, USA). The cDNA synthesis was performed with the CycleScript RT premix system (BIONEER, Deajeon, Korea). To amplify XBP-1 mRNA, PCR was performed for 35 cycles (94 °C for 30 s; 55 °C for 30 s; and 72 °C for 1 min (7 min in the final cycle)) using primers 5′-AAC TCC AGC TAG AAA ATC AGC-3′ (Forward) and 5′-CCA TGG GAA GGA TGT TCT GGG-3′ (Reverse) with PCR premix (BIONEER). Three fragments, representing spliced (sXBP-1, 215 bp), unspliced (usXBP-1, 241 bp) and hybrid (hXBP-1, 267 bp) were produced and detected by running on 3% agarose gels and staining with ethidium bromide. The composition of the primers used in the RT-PCR are the following: GRP78 Forward 5′-GTT CTT CAA TGG CAA GGA ACC ATC-3′, Reverse 5′-CCA TCC TTT CGA TTT CTT CAG GTG-3′; CHOP Forward 5′-GCA CCT CCC AGA GCC CTC ACT CTC C-3′, Reverse 5′-GTC TAC TCC AAG CCT TCC CCC TGC G-3′; GAPDH Forward 5′-TAG ACG GGA AGC TCA CTG GC-3′, Reverse 5′-AGG TCC ACC ACC CTG TTG CT-3′.

### 3.6. Plasmid

Based on the published sequence of the human GRP78 gene, a 378-bp fragment of the GRP78 promoter region (-371 to region; relative to the transcriptional initiation site) was amplified by PCR from genomic DNA with the following sets of primers and cloned into the KpnI and HindIII sites of the pGL3-basic vector (Promega), which contains the luciferase reporter gene: sense strand (5′-CGGGGTACCGTCACTCCTGCTGGA-3′) and antisense strand (5′-CATCCCAAGCTTTCGACCTC ACCGTCGCCTACTC-3′).

### 3.7. Western Blot Analysis

After different treatments, cells were washed three times with ice-cold phosphate-buffered saline (PBS) and collected by scraping in lysis buffer containing 50 mM Tris-HCl (pH 8.0), 150 mM NaCl, 1% Nonidet P-40 (NP-40), 0.5% sodium deoxycholate, 0.1% SDS, 1X protease inhibitor cocktail set III (Calbiochem, Darmstadt, Germany), 0.5 µM Cantharidin (Calbiochem), 1 mM Na_3_VO_4_ and 1 mM DTT. The mixture was disrupted by three freeze-thaw cycles and subsequently centrifuged at 15,000 rpm at 4 °C for 30 min. The supernatant was collected and preserved at −70 °C until use. Total protein was transferred to a polyvinylidene difluoride (PVDF) membrane and was subjected to western blot analysis with specific antibodies. Immune complexes were detected with enhanced chemiluminescence reagents.

## 4. Conclusions

Verrucarin A (**1**) was isolated from the culture broth of *Fusarium* sp. F060190. The compound showed potential inhibitory activity on tunicamycin-induced GRP78 promoter activity assay using FaO rat liver cells. Also, the compound decreased the gene expression of GRP78, CHOP, and XBP-1, and it reduced phosphorylation of IRE1α in FaO rat liver cells by tunicamycin. Therefore, these results provide evidence that verrucarin A is a potential ER stress regulator, and it could be useful candidate for therapeutic use in cases involving diabetes, obesity, and disorders related with dysfunction of ER-stress.
